# Immune Evasion of *Mycoplasma bovis*

**DOI:** 10.3390/pathogens10030297

**Published:** 2021-03-04

**Authors:** Hussam Askar, Shengli Chen, Huafang Hao, Xinmin Yan, Lina Ma, Yongsheng Liu, Yuefeng Chu

**Affiliations:** 1 State Key Laboratory of Veterinary Etiological Biology, Lanzhou Veterinary Research Institute, Chinese Academy of Agricultural Sciences, Xujiaping 1, Lanzhou 730046, China; Hussamaskar@azhar.edu.eg (H.A.); chenshengli@caas.cn (S.C.); haohuafang@caas.cn (H.H.); yanxinmin@caas.cn (X.Y.); malina@caas.cn (L.M.); liuyongsheng@caas.cn (Y.L.); 2 Faculty of Science, Al-Azhar University, Assuit 71524, Egypt

**Keywords:** *Mycoplasma bovis*, immune response, exhausted T-cell, infection, immune evasion

## Abstract

*Mycoplasma bovis (M. bovis)* causes various chronic inflammatory diseases, including mastitis and bronchopneumonia, in dairy and feed cattle. It has been found to suppress the host immune response during infection, leading to the development of chronic conditions. Both in vitro and in vivo studies have confirmed that *M. bovis* can induce proinflammatory cytokines and chemokines in the host. This consists of an inflammatory response in the host that causes pathological immune damage, which is essential for the pathogenic mechanism of *M. bovis*. Additionally, *M. bovis* can escape host immune system elimination and, thus, cause chronic infection. This is accomplished by preventing phagocytosis and inhibiting key responses, including the neutrophil respiratory burst and the development of nitric oxide (NO) and inducible nitric oxide synthase (iNOS) that lead to the creation of an extracellular bactericidal network, in addition to inhibiting monocyte and alveolar macrophage apoptosis and inducing monocytes to produce anti-inflammatory factors, thus inducing the apoptosis of peripheral blood mononuclear cells (PBMCs), inhibiting their proliferative response and resulting in their invasion. Together, these conditions lead to long-term *M. bovis* infection. In terms of the pathogenic mechanism, *M. bovis* may invade specific T-cell subsets and induce host generation of exhausted T-cells, which helps it to escape immune clearance. Moreover, the *M. bovis* antigen exhibits high-frequency variation in size and expression period, which allows it to avoid activation of the host humoral immune response. This review includes some recent advances in studying the immune response to *M. bovis*. These may help to further understand the host immune response against *M. bovis* and to develop potential therapeutic approaches to control *M. bovis* infection.

## 1. Introduction

**Figure 1:**
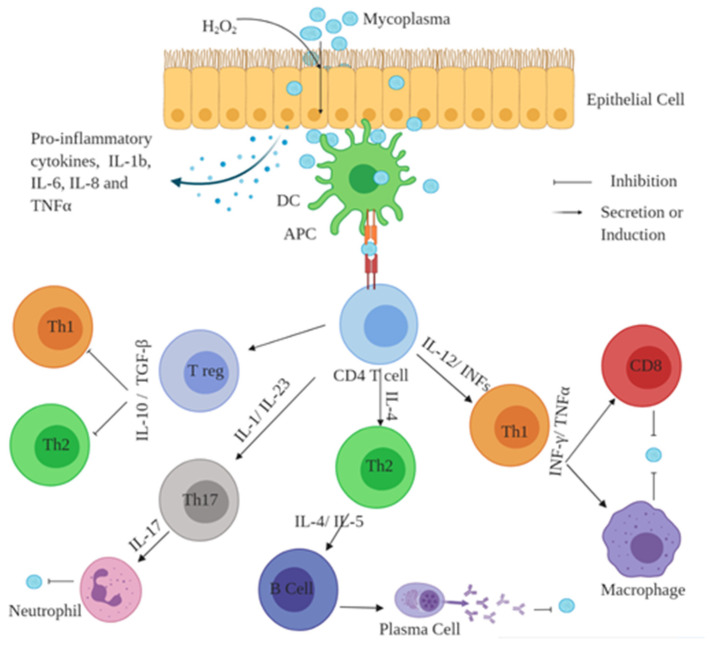
The production of hydrogen peroxide (H_2_O_2_) plays a crucial role in *M. bovis* virulence. *M. bovis* adheres to surfaces of bronchial epithelial cells that release various cytokines when exposed to *Mycoplasma* (created using BioRender.com).

Infection of dairy and feed cattle by *Mycoplasma* is a global issue with consequences for cattle health and economics. *M. bovis* is responsible for bronchopneumonia, pharyngitis, laryngitis, otitis, keratoconjunctivitis, meningitis, and mastitis, as well as hormonal diseases such as metritis, infertility, and abortion. All age groups are susceptible to *M. bovis* infection, and the pathogen can be shed and survive for years in contaminated herds [1]. Such diseases have substantial economic implications for the dairy and beef industries [2,3]. In the past few years, *M. bovis* has emerged as a significant pathogen in Europe [3], North America [4], and Japan [5], resulting in calf mortality, weight loss, and a significant drop in milk production [6]. The use of mycoplasma antibiotics is often ineffective and presents a global problem, as tetracycline- and spectinomycin-resistant bacteria are increasing in prevalence [7]. In addition, *M. bovis* prevents lymphocyte proliferation due to its immune-suppressive characteristics [8], and promotes bovine lymphocytic apoptosis in response to mitogens [9]. Although there have been several studies on *M. bovis* infections, both in vivo and in vitro, there are conflicting findings regarding whether these same processes take place during bona fide *M. bovis* infection. Several research assessments have revealed that *M. bovis* adheres to surfaces (Figure 1), is located between cells, and does not transfer intracellularly on bronchial epithelial cells [10]. Contrary to this, other studies show that *M. bovis* attaches to cell surfaces and migrates intracellularly into neutrophils and macrophages [11,12,13]; this occurs through phagocytosis via active neutrophils and macrophages. Furthermore, it can invade erythrocytes [14,15] when moving through the immune system [9]. *Mycoplasma* can spread systemically by invading peripheral blood mononuclear cells (PBMCs) and erythrocytes while evading immune reactions. Some studies have indicated the *M. bovis*-induced activation and development of different cytokines from bovine macrophages, such as IFN-γ, interleukin-4 [16], TNF-α, and nitric oxide [14]. This is not surprising, as *M. bovis* has been implicated by numerous studies (including those listed above) in the regulation of immune response both in vivo and in vitro [15,17]. Van der Merw et al., (2010) showed that *Mycoplasma bovis* strain Mb1 invades and adheres to bovine PBMCs, preventing their proliferation, but does not appear to alter developmental responses in functional cytokines such as IFN-γ. In a relatively short time, *M. bovis* can invade any type of PBMC, resulting in the induction of lymphocyte differentiation and spreading to any host tissue. Adhesion is the primary step of infection with mycoplasma [18]. A few recognized uncovered surface proteins have been classified as adhesins [19,20]. However, cell-dependent adhesion molecular pathways have not yet been analyzed in depth. α-Enolase, NADH oxidase (NOX), and methylenetetrahydrofolate-tRNA-(uracil-5-)-methyltransferase (TrmFO), which bind to fibronectin and plasminogen and function as the connection between the host cell receptors and bacterial adhesion, have recently been identified as adhesins [21,22,23] that may promote invasion and *M. bovis* dissemination in hosts [24]. In vivo defensive studies on the irregular intracellular positioning of *M. bovis* in host cells are available [12,25,26,27]. Inconsistent findings on PBMC contamination by *M. bovis* have been obtained regarding apoptosis induction, cytotoxic effects, and the host cells’ viability [8,28,29,30]. Similarly, *Mycoplasma bovis* has been found to inhibit the development of PBMCs [8,29,31,32].

Dudek et al., (2020) explored the leukocytes characterizing the host antimicrobial resistance mechanisms. Several in vitro experiments have been carried out on the effect of leukocytes on *M. bovis*; however, these findings are difficult to transfer to in vivo conditions. Moreover, only a few experiments have been carried out on the local immune reaction in *M. bovis*-induced pneumonia. Cytometry tests were carried out on an experimental calf infection model to estimate the effects of an *M. bovis* strain in the field on changes in the peripheral blood leukocyte reaction, including phagocytic activity and oxygen metabolism. Immunohistochemical staining has been used to assess the local lung immunity of experimentally infected calves. The general stimulation of phagocytic activity and the killing mechanism of peripheral blood leukocytes in response to *M. bovis* infection indicate the upregulation of cellular antimicrobial pathways. In infected lungs, local immune responses are characterized by T- and B-cell activation but with more enhanced lymphocytic T-response. The activation of local lung immunity has also been confirmed by the high expression of phagocytes and antigen-presenting cells post-infection. Stimulation does not seem to be effective in eliminating *M. bovis* from the host and preventing specific lung lesions, suggesting the pathogen’s ability to avoid the host immune reaction—either by peripherals or by local cells—in *M. bovis*-induced pneumonia [33].

This review discusses the displayed antigens of naive T-cells in dendritic cells (DCs) by large histocompatibility complex–T-cell receptor interaction, which causes antigen-specific T-cells to be activated. T-cells differentiate into four sub-types, such as T-cells Th1, Th2, and Th17, or regulatory T-cells (Tregs), based on the microenvironmental cytokine environment (involving IL-1, IL-4, IL-5, IL-6, IL-10, IFNs, IL-12, IL-17, IL-23, TNF-α, and TGF-β). Th1 cells activate macrophages or CD8 T-cells by releasing IFN-γ. CD8 T-cells either degrade pathogens or cause apoptosis of infected cells by producing perforins and granzymes. Th2 cells activate B-cells by synthesizing cytokines and separating B-cells from plasma cells, modifying the antibody class and promoting the affinity to antibodies involved in pathogenic neutralization, opsonization, and phagocytosis. In neutrophil activation and immune regulation, Th17 cells are involved in the production of IL-17A, which is essential for protection against extracellular and specific intracellular pathogens. Regulatory T-cells (Tregs) control the *M. bovis* immune responses and sustain self-tolerance by negatively delivering Th1 and Th2 cells by developing the cytokines IL-10 and TGF-β. 

## 2. The Immune Response to *M. bovis*

### 2.1. Humoral and Cellular Immunity

The immune response can be divided into humoral immunity and cellular immunity, with cellular immunity regulated by T-cells and B-cells regulating humoral immunity. The immune system develops cytokines, including interleukins, which balance humoral and cell-mediated immune responses. T-cells have a specific antigen (antibody)-binding receptor molecule on the cell surface, which is called a T-cell receptor (TCR). Many TCRs recognize parts of major histocompatibility complex (MHC) molecules, especially the antigenic peptides linked to those molecules. T-helper cells (Th CD4 cells) recognize processed foreign peptides on the surface of antigen-presenting cells (APCs) complexed with MHC class II molecules and induce an immune response by secreting cytokines that stimulate CD8 T-cells and B-cells. Killer or cytotoxic T-cells (CD8) participate in surface interactions with other cells carrying processed foreign peptides complexed with MHC class I molecules [13,34]. Cellular immunity also includes regulatory T-cells (T_reg_), which, after the immune response, suppress the development of CD8 T-cells and memory T-cells [35]. Immune response induction—both humoral and cell-mediated—depends on the activation of Th-cells and cytokines’ release [36]. In specialized antigen-presenting cells (APCs), including macrophages, dendritic cells, and B-cells, among others, antigens are recruited to process and present peptides on Th-cell class II molecules. Upon identifying the MHC class II complex, the Th-cells become activated, and their function changes to that of effector Th- and memory Th-cells [35]. The activated Th-cells secrete different cytokines, primarily IL-2, which functions autocrinally and increases the population of Th-cells. The cytokine production by Th-cells also initiates B-cells’ division into memory B-cells and the secretion of antibodies by plasma cells. T-helper cells also secrete cytokines, which drive T-cells to become memory T-cells and effector T-cells, cytotoxic T-cells that recognize and lyse modified self-cells, such as virus-infected cells recognized by the antigen supplied by MHC class I molecules [13]. It has been stated that the cellular immune response is more successful than the humoral response in the elimination of *M. bovis* from the host [37]. A critical event essential to the successful activation of the immune response to any infectious pathogen is the mechanism of antigen processing and presentation by MHC class I and II molecules. Both cell-mediated and humoral immune responses can be improved by vaccination to ensure that *M. bovis* infiltrating mucosal sites are eliminated before the onset of the disease.

### 2.2. Mucosal Immunity

The mucosal immune system includes digestive, gastrointestinal, urogenital, and exocrine glands. The predominant isotype formed in bovine species at mucosal sites is IgG1. The antigen-specific immune responses of Peyer’s patches dictate mucosal immune development through the production of cytokines following immune cell interactions. Since oral and transtracheal inoculation of *M. bovis* mainly colonized the upper respiratory tract and the tonsillar mucosa, at the autopsy, both colonized the palatine and pharyngeal tonsils with *M. bovis* in all inoculated calves. The tonsils of orally inoculated cattle had the largest amounts of *Mycoplasma*. These results confirm previous reports of naturally occurring *M. bovis* infection in calves indicating the initial colonization site in the upper respiratory tract [38]. Consequently, lymphoid tissue, particularly antibodies, is necessary to localize mycoplasma infections at mucosal sites of disease, preventing transfer to other tissues and arthritis production [35]. With high IL-4 levels and low levels of IFN-γ and IgG1 antibodies, the pulmonary bovine immune response to *M. bovis* is primarily anti-inflammatory. Simultaneously, it was found that *M. bovis* primarily infects the mucosal and serosal surfaces and live bacteria in infected cattle brains. Previous studies saw bacterial antigens also in the synovial membranes and infected cattle’s liver and kidneys [9]. Different strains of *M. bovis* reside on mucosal and extracellular surfaces but can invade and survive in various host cells. *M. bovis* can be seen living within the bovine alveolar macrophages. *M. bovis* infection in bovine epithelial cells occurs in many epithelial cell types, including embryonic bovine tracheal cells, embryonic bovine lung cells, and primary bovine mammary cells [39]. *M. bovis* typically produces a heavy local mucosa characterized by high serum IgG and IgA reactions. Similarly, *M. bovis* mammary gland inoculation results in IgG serum and local IgG and IgA serum mucosa. However, to prevent infections, systemic antibodies are critical, and serum IgG *M. bovis* titers are associated with arthritis defense. Local antibodies are likely to be more critical on mucosal surfaces. For example, concentrations of anti-*M. bovis* antibodies correlated in milk but not in serum with resistance to reinfection in cows after *M. bovis* mastitis. IgG concentrations bronchoalveolar lavage fluid (BAL) are linked to resistance to respiratory disease associated with *M. bovis*. *Mycoplasma* respiratory infections are commonly recognized to have significant immunopathological components, characterized by large accumulations of lymphocytes in infected tissues, cytokine output, and inflammation of the lungs [2]. *Mycoplasmas*, including *M. bovis*, may also modulate some inflammatory responses. However, in the lungs of calves with *M. bovis* infections, little is known about the cytokine environment. In response to the *M. bovis* antigen, peripheral blood mononuclear cells from *M. bovis*-infected calves secreted IFN-γ and IL4 and developed a strong systemic IgG1 response with little IgG2. These results suggest that *M. bovis* induces a mixed Th1-Th2 cytokine response, although the lack of development of IgG2 was more consistent with a Th2-biased response [2]. The strategies adopted by pathogens during infection fall into two different categories: those that alter the host immune response and those that cause the pathogens to transform themselves or their location in the host to escape the host immune response.

## 3. Strategies Associated with the Host

### 3.1. Escape from Innate Immune Response 

**Figure 2:**
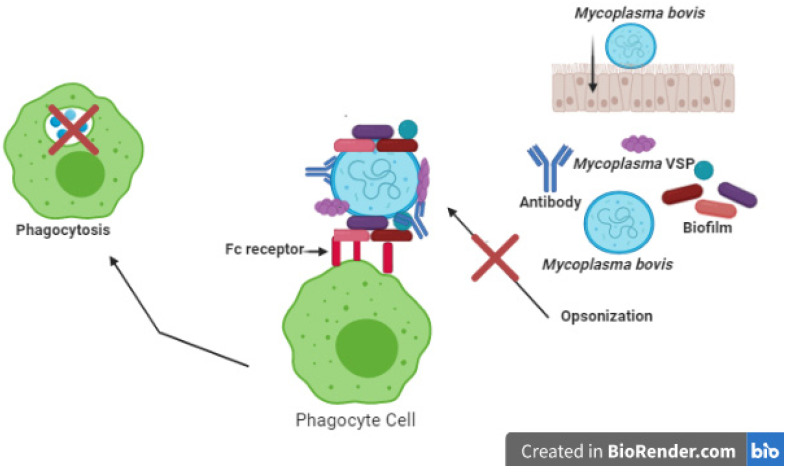
*Mycoplasma bovis* affects opsonization (created using BioRender.com).

Innate defense mechanisms are activated to fight infections. The innate immune system involves the skin, mucosal surfaces, and other components, including macrophages, dendritic cells, neutrophils, and natural killer cells. Extracellular pathogens are first phagocytized by macrophages and neutrophils and then eliminated. Gamma delta T-cells (γδ T-cells) are associated with immunization against bovine respiratory infection. Early response cytokines, including interferons (IFNs) and those of the complement system, are other vital components of the innate immune system [13]. *M. bovis* blocks the ability of neutrophils to produce a chemiluminescent reaction after certain species have been ingested [17]. It has been noted that *M. bovis* inhibits the blasts of respiratory neutrophils at or distal to the point of protein kinase C activation. A direct interaction between *M. bovis* cells and the neutrophil membrane is thought to be mediated by this effect; thus, it is essential to investigate the exact inhibition mechanism as well as the association between *Mycoplasma* spp. and innate immune cells typically containing proinflammatory cytokines such as IL-1, IL-6, and TNF-α. Furthermore, chemokines such as MIP-lα, MIP-1β, and ENA-78 are also produced through the evolution of IL-1, IL-6, and TNF-α; these, along with other epithelial and fibroblast cells, support other innate cells and adaptive immune cells. Chemokines contribute to the normal perivascular and peribronchiolar accumulation of mononuclear cells during mycoplasma respiratory infections [40]. The function of the dendritic cell when fighting mycoplasma infections is unknown. Incubation of *M. bovis* using alveolar macrophages led to the production of two essential mediators of the immune response: NO and TNF-α. Antigens derived from *M. bovis* can also activate bovine γδ T-cells in vitro. Although T-cells are traditionally called adaptive immune cells, it has been recognized that in cattle with innate immunity, peripheral γδ T-cells are regularly activated (e.g., as with mycobacterial antigens [41], which can present antigens to Th-cells). The role of bovine γδ T-cells in innate immunity in vivo has not yet been elucidated. Innate immune cells triggered by *M. bovis* have been reported, but it is not yet known whether Toll-like receptors (TLRs) are involved in such *M. bovis* triggers. Compared to macrophages, neutrophils seem to be more inhibited by *M. bovis* [40]. Efficient phagocytosis of *M. bovis* by bovine neutrophils and macrophages is based on opsonization [42]. *M. bovis* has various mechanisms to combat opsonization (Figure 2): First of all, the antigen difference on the surface is guided, at least in part, by selection pressures [43] induced by antibodies—an essential tactic to evade antigens. Second, *M. bovis* can provide physical defense against opsonization and phagocytosis through biofilm formation [44]. Third, two proteins that damage immunoglobulins, one that binds IgG (*Mycoplasma* Ig-binding protein; MIB) and another that clings to IgG heavy chains, are expressed in some forms of mycoplasmas (*Mycoplasma* Ig protease; MIP) [45]. Although this mechanism has not been tested in *M. bovis*, several copies of the MIB and MIP genes have been entirely sequenced in all *M. bovis* genomes [46]. This mechanism provides *M. bovis* with a different strategy to resist antimicrobial phagocytosis (Figure 2).

Opsonization of the antibody or substitution considerably improves phagocytic bacteria’s chances being destroyed by phagocytic cells (e.g., macrophages and neutrophils). *M. bovis* has many pathways to resist opsonization, thereby preventing and eliminating phagocytosis. 

Innate immunity is available or easily induced in a natural manner; however, these components are not antigen-specific and, more importantly, there is no immunological memory. On the other hand, memory’s extraordinary property through adaptive immunity has been demonstrated [13]. Initially, the adaptive immune cells responded to *M. bovis* after an initial response of innated cells during *Mycoplasma* infection [47].

### 3.2. Escape from Adaptive Immune Response 

Cells that come into contact with *Mycoplasma* often release cytokines known for their adaptive immune response, such as IL-13, to assess the type of adaptive immune response that occurs during *M. bovis* infection [40]. The humoral immune response to *M. bovis* seems skewed against IgG1, a less effective phagocytosis challenger and neutrophil killing than IgG2 [42,48,49,50,51]. *M. bovis* is an infectious organism that affects the host in both an immunostimulatory and immunosuppressive manner. Previous studies have shown an improvement in the percentage and subsets of T lymphocytes (i.e., CD4 and CD8 cells) in reaction to *M. bovis* experimental challenge in calves. Leukocytosis and a substantial increase in the percentage of WC4 (B lymphocytes) have also been observed under these conditions, suggesting the activation of CD4, CD8, and γδ T-cells due to *M. bovis* antigen incubation [34]. Blastogenesis and serological measures have been used to assess the immune response to *M. bovis*; however, *M. bovis* antigen levels of proliferation are not pronounced and have been absent in some studies. Aside from these variables and serological experiments, serology assessment has been used for the determination of the adaptive immune response to *M. bovis*. In lung disease, the immune response to *M. bovis* infection has been biased towards a type-2 immune response by analyzing peripheral lymphocytes and serum (classically considered a humoral response). Immunohistochemical studies have suggested that many IgGl cells are generated, consistent with serological trials (IgGl indicates a Th2 response in infected cattle’s lungs) [47]. The infection by *M. bovis* continues to proceed as a chronic infection in terms of both the innate and adaptive immune cells, with a rebound in post-clinical animals for several months. It has been speculated that one of the reasons facilitating the chronic nature of bovine mycoplasma infection is that the serological reaction may not be that of the right phenotype, such as IgM. In addition, IgGl is an inferior complement activator relative to other IgG isoforms [47] such as IgG2.

A previous study found that *M. bovis* could evade the host immune response. *M. bovis* has immunosuppressive properties that prevent lymphocyte proliferation in response to mitogens [52].

Lymphocyte proliferation inhibition has been seen as a supposed mechanism for immune evasion by *M. bovis* [10]. *M. bovis* has been shown to be active in proliferative blood lymphocytes under mitogen inhibition [15]. DNA synthesis may be observed in cells already activated by mitogens. Additionally, a peptide released by *M. bovis* was reported to suppress the naive activation of lymphocytes [29]. It has been shown that this peptide is manifested during bovine lung *Mycoplasma* infections, indicating that this lymphocyte-inhibiting peptide may act as a mediator of immune evasion. Vanden et al. [29] also stated that *M. bovis* is responsible for, or causes, cell apoptosis. *M. bovis* has been identified as a pathogen responsible for the development of respiratory and arthritis diseases. There is a mass infiltration of lymphocytes and non-specific activation in arthritis, with no death of lymphocytes. Therefore, these authors indicate that *Mycoplasma* might not cause T-cell apoptosis. The apoptotic effects do not inhibit the infiltration of T-cells or cytokine production in other cell types [29]. 

### 3.3. Cytokine Release during M. bovis Infection

*M. bovis* can induce the expression of important cytokine (TNF-α, IL-1 β, IL-18, IL-2, IL-12, IFN-γ, and IL- 6) genes in bovine PBMCs at a multiplicity of infection (MOI) of 1000, as recorded by Gondaira et al. [30]. These findings suggest that disease could result from small numbers of bacteria low enough not to trigger an immune reaction, in contrast to large numbers of *M. bovis*, which would engage the immune response. The upregulation of IFN-γ, which is essential for host immunity against intracellular pathogens, has been demonstrated by IL-12. Thus, IL-12 and IFN-γ play crucial roles in *M. bovis* infection. Previous studies have shown that heat-killed *M. bovis* did not induce expression and cytokine production of bovine PBMC cytokine mRNA, and have indicated the potential of live *M. bovis* to play a pathogenic role in the bovine PBMC immune response [30]. TNF-α is a proinflammatory cytokine that encourages endothelial activation and leukocyte recruitment at the injection site [53]. Previous research has shown that *Mycoplasma bovis* induction was delayed by the inflammatory response [54]. *Mycoplasma bovis* has been detected in natural milk secreted following *M. bovis* intramammary (IMI) infusion (10^6^ to 10^10^ CFU/mL) for days prior to the appearance of clinical signs [55]. Previous experiments have demonstrated elevated levels of IL-12 and IFN-γ in milk after *M. bovis* intramammary infusion [56]. *M. bovis* was intracellularly detected in bovine PBMCs [8]. IL-12, which is essential for host immunity to intracellular pathogens, has been shown to upregulate IFN-γ [57]. As effectors of antigen-presenting cell (APC) function, inflammatory cytokines mobilize the immune system in response to danger and increase the efficiency of the immune response [58]. This can play a significant role in the biological response of bovine neutrophils to bacterial infections [59,60]. Rodriguez et al., (2015) noted the enhanced expression of TNF-a, IL-4, IL-10, and IFN-γ in the lungs of cattle infected with *M. bovis*. Significant variations in the expression of IL-1α, IL-1β, IL-2, IL-6, and IL-8 were observed between infected vs. normal lungs of control calves [61]. The cells activated by *M. bovis* can release cytokines [14]. This is also important in pathogenesis as TNF-α and IL-12 can be produced in vitro by neutrophils after *M. bovis* infection for Th1 reaction [62]. *Mycoplasma bovis* delays apoptosis in monocytes and suppresses the development of IFN-γ and TNF-α [9], which promote its persistence and systemic diffusion through effective immunomodulation. *M. bovis* infection resulted in increased expression and a comparatively lower IL-1 response of the proinflammatory cytokine IL-6. Proinflammatory cytokines are essential for *M. bovis* pathogenesis. Although IL-6 was upregulated following co-incubation of PBMCs with live *M. bovis* at 100:1 MOI, there was no increase in IL-1 and TNF-α levels. It can be assumed that a high MOI is required for producing a consistent inflammatory response [63].

### 3.4. Immune Response Exhaustion during M. bovis Infection 

**Figure 3:**
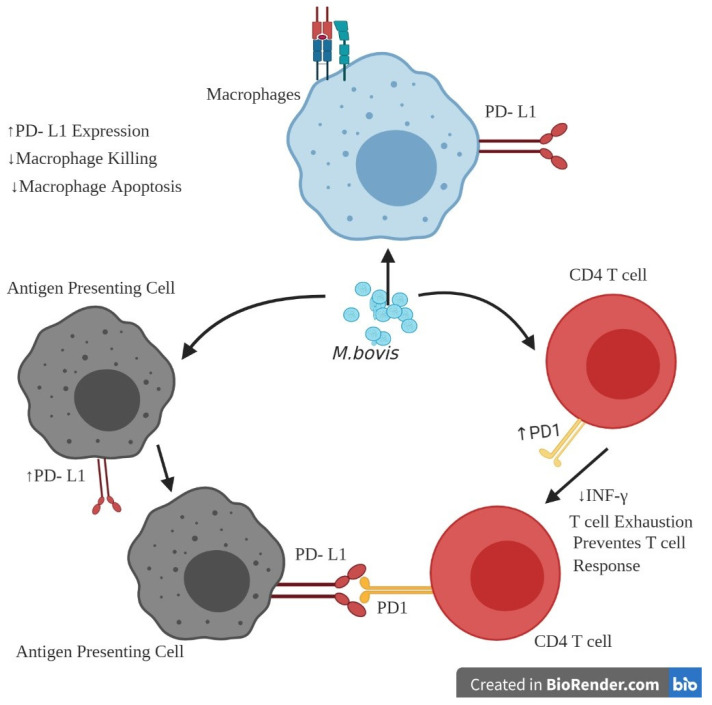
Induction of PD-1 and/or PD-L1 in *M. bovis* immune suppression. The receptor PD-1 (programmed death) is usually expressed at low concentrations with T-cell activation. Increased PD-1 levels occur in T-helper and cytotoxic T-cells. *Mycoplasma bovis* also increases the expression of programmed death-ligand 1 (PD-L1) in antigen-presenting cells and macrophages. PD-L1 in macrophages reduces macrophage killing of the bacteria and avoids cell apoptosis. The co-expression of PD-1 on T-cells and PD-L1 on antigen-presenting cells reduces T-cells’ potential for both replication and destruction, contributing to T-cell exhaustion (created using BioRender.com).

In chronic infection, immunosuppressive factors such as programmed cell death 1 (PD-1), lymphocyte activation gene 3 (LAG3), cytotoxic T-lymphocyte-associated protein 4 (CTLA4), T-cell immunoglobulin, and mucin-containing domain-3 (Tim3), are expressed in lymphocytes. These proteins bind to their respective ligands to cause immune suppression of the effector cells. These immune exhaustion-associated proteins cause a marked decrease in cell proliferation and cytotoxic activity. Newly identified *M. bovis*-induced mastitis has increased expression of immune exhaustion-related genes and decreased expression of innate mononuclear cells (MNC)-related immune response genes in cow quarters. These results showed that the immune function of bovine mononuclear cells (MNCs), linked to a prolonged duration of infection with *M. bovis*, was impaired by mastitis caused by *M. bovis* [52]. Based on these interpretations, it is essential to explain the associated processes more fundamentally. In a recent study, increased IFN-γ output by PD-1/PD-L1 blockades in bovine mycoplasmosis was disclosed [64], and many examples were observed for infections involving numerous other pathogens, such as *Anaplasma marginale* [65], *Plasmodium berghei* [66], *Mycobacterium avium* subsp. *paratuberculosis* [65], and bovine leukemia virus [67,68]. Suleman et al., (2018), using flow cytometry (FCM) and immunofluorescence tissue staining, showed that the expression of PD-L1 on macrophages in bovine tracheal (EBTr), bovine lung (BLL), and bronchoalveolar lavage (BAL-MØ) cells was increased following *M. bovis* infection. The differently modulated subpopulations of CD8 and CD4 T-cells with intermediate and robust expression of PD1 after *M. bovis* infection of cattle PBMC have also been studied. T-cells showed a substantial decrease in CD4 PD1 and CD8 PD1 subsets and a significant increase in CD4 PD1 and CD8 PD1 subsets. Furthermore, the use of blocking PD-1 antibodies results in the restoration of *M. bovis* strain Mb1 after PD-1/PD-L1 destruction as well as the proliferation of *M. bovis* strain Mb1 in contaminated PBMCs [31]. A recent study showed that PD-1/PD-L1 (Figure 3) is important for *Mycoplasma bovis* pathogenesis and related immune exhaustion in inhibitory pathways, resulting in compromised host immune responses [31]. Previous studies indicated that the development of Th1 cytokines (i.e., IFN-γ and TNF-α) by bovine leukemia virus (BLV)-specific T-cells of infected cattle was reactivated by the blockade PD-1 and LAG-3 pathways. These findings show that PD-1 and LAG-3 mediate the functional exhaustion of CD4 and CD8 T-cells cooperatively and are associated with B-cell lymphoma development in cattle infected with bovine leukemia virus (BLV) [69].

## 4. Conclusions

This review summarized some recent advances in the immune response relating to *M. bovis* infection and immunity, as well as the strategies by which *M. bovis* evades the innate and adaptive immune response, immune response exhaustion, and cytokines in addition to the proliferative response of bovine PBMC cells to *M. bovis*. *M. bovis* induces the host to produce an inflammatory response, causing pathological immune damage—its essential pathogenic mechanism. From the current literature, it can be concluded that *M. bovis* can induce the host to produce γδ, CD4, and CD8 T-cells as well as influence the host to produce IgM, IgG, and IgA antibodies in a time-dependent manner. Compared with IgG2, the IgG1 produced by the host induced by *M. bovis* is dominant. The IgG1 antibody has a low opsonin effect, leading to an inadequate humoral immune response and long-term infection of *M. bovis* in the host. *Mycoplasma bovis* can cause a wide variety of immune-modulatory products by directly activating cytokine secretion from lymphocytes, macrophages, and neutrophils. Surface antigen variation, potential intracellular invasion, and biofilm formation also allow *M. bovis* to impair the host immune response. However, further research on the host and microbial factors regulating *M. bovis* infection in the upper respiratory tract, contributing to disease development or the effective immune response, is required to facilitate the creation of better control mechanisms and preventative strategies for the pathogen. Further studies of host immune responses to *M. bovis* infection are needed to control the resulting disease. 

## Data Availability

Not applicable.
